# The Economic Burden of Non-fatal Musculoskeletal Injuries in Northeastern Tanzania

**DOI:** 10.5334/aogh.1355

**Published:** 2019-03-04

**Authors:** Sonya Davey, Evgeny Bulat, Honest Massawe, Anthony Pallangyo, Ajay Premkumar, Neil Sheth

**Affiliations:** 1 Perelman School of Medicine at the University of Pennsylvania, Philadelphia, US; 2 Department of Orthopaedics and Traumatology, Killimanjaro Christian Medical Center, Moshi, TZ; 3 Department of Orthopaedic Surgery, Hospital for Special Surgery, New York, US; 4 Department of Orthopaedic Surgery, Hospital of the University of Pennsylvania, Philadelphia, US

## Abstract

**Background:**

Although musculoskeletal injuries have increased in sub-Saharan Africa, data on the economic burden of non-fatal musculoskeletal injuries in this region are scarce.

**Objective:**

Socioeconomic costs of orthopedic injuries were estimated by examining both the direct hospital cost of orthopedic care as well as indirect costs of orthopedic trauma using disability days and loss of work as proxies.

**Methods:**

This study surveyed 200 patients seen in the outpatient orthopedic ward of the Kilimanjaro Christian Medical Center, a tertiary hospital in Northeastern Tanzania, during the month of July 2016.

**Findings:**

Of the patients surveyed, 88.8% earn a monthly income of less than $250 and the majority of patients (73.7%) reported that the healthcare costs of their musculoskeletal injuries were a catastrophic burden to them and their family with 75.0% of patients reporting their medical costs exceeded their monthly income. The majority (75.3%) of patients lost more than 30 days of activities of daily living due to their injury, with a median (IQR) functional day loss of 90 (30). Post-injury disability led to 40.6% of patients losing their job and 86.7% of disabled patients reported a wage decrease post-injury. There were significant associations between disability and post-injury unemployment (p < .0001) as well as lower post-injury wages (p = .022).

**Conclusion:**

This exploratory study demonstrates that in this region of the world, access to definitive treatment post-musculoskeletal injury is limited and patients often suffer prolonged disabilities resulting in decreased employment and income.

## Introduction

In 2008, Paul Farmer and Jim Kim stated, “In Africa, surgery may be thought of as the neglected stepchild of global public health [1].” Africa has the highest rate of surgical disability adjusted life years (DALYs) due to injuries of any continent [2]. Disease burden associated with surgical conditions is estimated as 38 DALYS lost per 1,000 population in Sub-Saharan Africa and this value is primarily due to injuries [3]. By 2020, it is projected that injury, including road traffic accidents, will contribute to 20% of the global burden of disease [3]. It has been reported that 32% more deaths result from injuries than from HIV/AIDS, tuberculosis, and malaria combined [4], and nearly half of all injury-related mortality occurs in individuals between the ages of 15 and 44 [5].

The global health community had largely neglected surgical diseases when supporting health interventions in the region due to a legacy misperception of high associated cost and thus likely poor cost-effectiveness. However, numerous studies have shown that essential surgery has a favorable cost-effectiveness profile when compared to more traditional interventions. For example, while it costs $19–102 per DALY averted for surgical treatment in district hospitals, oral rehydration therapy for diarrheal disease can cost $1062 per DALY averted and HAART therapy for HIV can range from $350–1494 per DALY averted [3]. Similarly, a recent meta-analysis demonstrated that the $381 per DALY averted for orthopedic surgery is lower than medical treatment for ischemic heart disease ($500–706 per DALY averted) and HAART treatment ($453–648 per DALY averted) [6].

The World Health Organization Global Initiative for Emergency and Essential Surgical Care was launched in 2005 and the 68th World Health Assembly adopted a resolution that recognized essential surgical care as part of universal health coverage in 2015 [7,8]. Although improving access for such surgeries has been considered a goal, access to essential surgeries for musculoskeletal injuries remains limited, largely due to total hospital costs, which are often shifted to out of pocket patient costs in sub-Saharan Africa. A study incorporating data from 15 African countries found that 50% of households financed out-of-pocket expenditure from hospitalizations by borrowing or selling assets [9]. The World Health Assembly, as well as independent organizations such as the Bellagio Essential Surgery Group, recommends that research on the socioeconomic impact of surgically correctable injuries at the district and country level is critical to developing informed policy and appropriate financing strategies in sub-Saharan Africa [3,9]. However, the burden of disability from injuries is poorly available in the region [10].

It is critical that studies examining the socioeconomic burden of injuries not only focus on hospitalization and surgery (direct costs), but also loss of work and productivity (indirect costs). McIntyre et al. found that the majority of studies analyzing healthcare costs in LMICs estimate indirect costs to be 2 to 3.6-times greater than direct costs [11]. Long-term post-fracture disability is prevalent in sub-Saharan Africa, with the incidence of permanent disability due to traumatic injury estimated at 50-times higher than mortality rate [12]. However, few studies have examined both direct and indirect costs associated with injury in this region.

The purpose of our study is to examine the socioeconomic impact of musculoskeletal injury at one of the largest tertiary hospitals in Tanzania. In addition, we performed a meta-analysis of all studies that examined costs associated with injury in Sub-Saharan Africa and we hypothesized that patient reporting of catastrophic expenditure (direct costs) and disability and wage loss (indirect costs) in Northern Tanzania will be similar to that of other regions within sub-Saharan Africa.

## Methods

### Setting

The study was conducted at Kilimanjaro Christian Medical Center (KCMC), located in Moshi, Tanzania. KCMC is the third largest hospital in the country and is the only tertiary referral hospital serving a population of 11 million people living in the districts of Arusha, Kilimanjaro, Tanga, Singida, and Manyara [13]. IRB approval for the study was obtained from KCMC.

### Study Design

We prospectively collected data from all patients visiting KCMC’s outpatient orthopedic clinic during the month of July 2016. The orthopedic clinic operates two days per week and treats an average of 532 patients per month [13].

A survey was created in English and then translated into Swahili by Tanzanian physicians that were bilingual. The survey was then tested by three local participants for readability and understanding prior to administration. All patients presenting to the orthopedic clinic were solicited for participation. Of the patients solicited, 90% consented and participated in the survey. Patients were informed that participation incurred no benefit or harm to their treatment, and were encouraged to ask questions as they filled out the survey. Surveys were collected at the end of each clinic day, and were anonymously placed in a folder by each patient. A total of 200 patients who presented with injuries from various dates of initial injury presentation were surveyed and included in our analysis.

### Data Collection

Patients completed paper surveys which were then coded into a Microsoft Excel-compatible, password protected, spreadsheet using Ona Software by a research associate fluent in Swahili.

### Statistics

Chi-Square and Student’s t-test were used to assess differences between various comparison groups and means, respectively. The statistical significance level, *alpha*, was adjusted to 5% (0.05). Continuous variables are reported as median and interquartile range (IQR) as defined as the difference between the 75th percentile and the 25th percentile. Categorical variables are reported as a proportion (percent) of the total surveyed. Work loss calculations were completed using monthly wage prior to injury and days of functionality lost.

## Results

### Patient Characteristics

The median (IQR) age of patients was 47 (28%) and 113 (56.3%) of the survey participants were male (Table 1). The survey population was significantly older than the Tanzanian national demographic, which has a median age of 17.7.

**Table 1:** Sex, Age.

Sex (n = 192)	Study Data *n (%)*	National Demographics

Male, n (%)	108 (56.3%)	49.4%
Female, n (%)	84 (43.8%)	50.6%
**Age (n = 195)** Median (IQR)	47 (28)	17.7
0–14	12 (6.2%)	43.7%
15–24	27 (13.8%)	19.9%
25–54	89 (45.6%)	29.9%
55–64	38 (19.5%)	3.5%
65+	29 (14.9%)	3.0%

Of the patients completing the survey, the majority had only a primary school education, similar to the median education levels in Tanzania, which is 8 years. The most common reported occupations were farmer 52 (32.9%), business employee or office-based profession 39 (24.7%), and student 18 (11.4%). Other reported occupations included teacher, laborer, driver, and police officer (Table 2). Our study had significantly fewer farmers (32.9%) and more service professionals than the national demographics in which 66.9% of Tanzanians are farmers.

**Table 2:** Education, Occupation.

Education (n = 188)		
	
None	6 (3.2%)	
Primary (8 years)	101 (53.7%)	
Secondary (12 years)	49 (26.1%)	
Above Secondary (12+ years)	32 (17.0%)	
**Occupation (n = 158)**		
	
Farmer	52 (32.9%)	
Business/Office-based profession	39 (24.7%)	
Student	18 (11.4%)	
Retired	6 (3.8%)	
Teacher	12 (7.6%)	
Laborer	6 (3.8%)	
Driver	8 (5.1%)	
Police Officer	1 (0.6%)	
House wife	3 (1.9%)	
Unemployed	11 (7.0%)	
Prisoner	2 (1.3%)	
Total	158	
**Occupation (n = 158)**	**Study Data *n (%)***	**National Demographics**

Agriculture	52 (32.9%)	66.9%
Industry & Services	106 (67.1%)	33.1%

Of the surveyed patients, 26 (28.6%) participants wrote that they had no monthly income as they were not employed. Of those with a monthly income, 196 (88.8%) reported a monthly income of less than $250. Only 4 patients (2.2%) earned over $500 per month. The median (IQR) monthly income of employed participants were $92 (43.12) (Table 3).

**Table 3:** Monthly Income Pre-Injury.

Monthly Income Pre-Injury (n = 92)	
No Income	26 (28.6%)
<$50	19 (20.9%)
$51–$100	20 (22.0%)
$101–$250	16 (17.4%)
$251–$500	9 (9.9%)
>$500	2 (2.2%)

### Injury Presentation

Approximately 40 (19.3%) patients presented with injuries that occurred less than two months prior to the survey, while 33 (16.5%) patients presented with injuries that occurred over 10 years ago (Table 4). Nearly 139 (69.2%) patients presented with lower extremity injuries.

**Table 4:** Presentation of Injury.

Injury Type (n = 183)	
Chronic Condition	72 (39.3%)
Sub-Acute/Acute Injury	103 (56.3%)
**Date of Injury (n = 176)**	
0–2 months ago	34 (19.3%)
3–6 months ago	34 (19.3%)
7–12 months ago	33 (18.8%)
13 months–4 years ago	28 (15.9%)
5 years–9 years ago	18 (10.2%)
10+ years ago	29 (16.5%)
**Location of Injury (n = 198)**	
Neck	4 (2%)
Spine	21 (10.6%)
Upper Extremities	22 (11.1%)
Hip	14 (7.1%)
Isolated Lower Extremities	94 (47.5%)
Polytrauma involving Lower Extremities	43 (21.7%)

Chronic condition is defined as >6 months; Sub-acute/acute is defined as <6 months.

### Indirect costs: Disability & Loss of Work

There were 100 (52.9%) patients who were able to independently perform activities of daily living (ADLs) prior to their injury, but were no longer able to do so post-injury, and were classified as suffering disability (Table 5). Activities that we used to describe ADLs included showering, walking, shopping, lifting a bucket, and cooking. There were 73 patients (75.3%) who lost greater than 30 days of ability to perform ADLs due to their injury; the median (IQR) days of inability to perform ADLs post-injury was 90 (30) (Table 5).

**Table 5:** Characteristics of Injured Patients–Disability and Loss of Work.

Patient could complete daily tasks for living prior to injury (n = 194)	
Yes	189 (97.4%)
No	5 (2.6%)
**Patient could complete daily tasks for living post – injury (n = 194)**	
Yes	77 (39.7%)
No	117 (60.3%)
**Employment status before and after injury (n = 175)**	
Previously working and did not have to change job	38 (21.7%)
Previously working and had to change job due to injury	6 (3.4%)
Previously working and lost job due to injury	71 (40.6%)
Never worked	60 (34.3%)
**Decrease in income post-injury (n = 104) (for those who have ever worked)**	
Yes	87 (83.7%)
No	17 (16.3%)
**Days of functionality lost due to injury (n = 97)**	
<10	11 (11.3%)
10 to 30	13 (13.4%)
31 to 60	21 (21.6%)
3 to 5 months	20 (20.6%)
6 months to 1 year	23 (23.7%)
>1 year	9 (9.3%)

The indirect cost of loss of work was calculated using monthly wage prior to injury and days of functionality lost. The average work loss cost from injury was $627.05 (95% CI: $333.36, $920.75) (Table 6).

**Table 6:** Average Medical and Work Loss Costs from Injury.

	Mean (95% Confidence Interval)

Medical Costs (n = 83)	$451.20 ($330.35–$572.05)
Work Loss Cost (n = 47)	$627.05 ($333.36–$920.75)
Combined Cost	$1,078.25

Post-injury disability led to 40.6% (71) of patients losing their job and 3.4% (6) changing their job. Overall, 87 (86.7%) disabled patients reported a wage decrease post-injury (Table 5). There were significant associations between disability and post-injury unemployment (p < .0001) as well as lower post-injury wages (p = .022) (Table 7).

**Table 7:** Comparing Disabled and Abled Patients.

	Disabled	Not disabled	p-value

Received Treatment	62/103 = 60.19%	25/63 = 39.68%	0.01
Currently Working	6/113 = 5.3%	38/73 = 52.0%	<.001
Received Other Care	73/93 = 78.5%	48/76 = 73.0%	.028
Has Insurance	51/112 = 45.5%	48/76 = 63.2%	.018

### Direct costs

The average out-of-pocket medical cost for patients presenting to KCMC was $451.20 (95% CI: $330.35, $572.05) (Table 6). To evaluate the direct cost burden of hospital costs on households, we used two outcomes measures: (a) the difference between monthly income and cost of treatment (Monthly Income – Healthcare Cost) (Table 5) and (b) patient perceived catastrophic burden. Of the 44 patients who reported direct healthcare costs and monthly income, 33 (75%) reported that their direct healthcare costs exceeded their monthly income and only 2 (4.5%) reported a monthly income $250 greater than their healthcare costs (Table 8).

**Table 8:** Difference between monthly income and hospital cost. (n = 44).

Less than –$250	17
–$250 to $0	16
$1 to $250	9
Greater than $250	2

Regarding perceived burden, we found that 179 (73.7%) patients reported that their injury healthcare costs are a catastrophic burden to themselves and their family.

To analyze the impact of health insurance on out-of-pocket costs, we stratified patients by access to health insurance (Table 9). While patients with health insurance paid an average of 20% less than patients without health insurance, the data demonstrate that there is not a statistically significant decrease in out-of-pocket costs with health insurance (p = .26). We analyzed insurance based on occupations and found that salaried people were not significantly more likely to have insurance in comparison to unsalaried people (p = .37) (Table 10). Overall, patients had either government or private insurance and type of insurance was not specified in the study.

**Table 9:** Comparing healthcare costs between patients with and without insurance. (n = 82).

	Total cost (mean, median)

Insurance (n = 24)	$376.82 ($545), $195.50 ($92)
No Insurance (n = 58)	$473.94 ($545), $322 ($123)

No significant difference, p = .26.

**Table 10:** Comparing occupation & health insurance.

Occupation (n = 158)	Insurance	No Insurance

Farmer (n = 52)	28	24
Business/Office-based profession (n = 39)	18	21
Student (n = 18)	9	9
Retired (n = 6)	5	1
Teacher (n = 12)	11	1
Laborer (n = 6)	4	2
Driver (n = 8)	2	6
Police Officer (n = 1)	1	0
House wife (n = 3)	2	1
Unemployed (n = 11)	5	6
Prisoner (n = 2)	0	2
Total (n = 158)	85	73
**Occupation (n = 158)**	**Insurance**	**No Insurance**

Salaried (n = 124)	69	55
Unsalaried (n = 34)	16	18
Total (n = 158)	85	73

No significant difference, p = .37.

## Discussion

Several orthopaedic surgical services, especially those related to orthopaedic trauma and emergency surgery, have been determined to be very cost-effective, and several are explicitly listed by the WHO as essential to prevent long-term disability or mortality [14,15]. However, both direct and indirect costs associated with surgical services remain a major barrier to patient access in these and similar settings [16].

In Tanzania, following the introduction of user fees at public hospitals in the early 2000s, out-of-pocket expenditures have become prevalent in all public hospital settings. Approximately 52% of Tanzania’s total health expenditure is out-of-pocket [4]. A study assessing adult multistage data from the Tanzania National Panel survey found that out-of-pocket costs are a serious obstacle to accessing healthcare among lower income laborers [17]. The report highlights the importance of analyzing out-of-pocket costs associated with accessing healthcare in Tanzania. Such analyses are particularly necessary in the context of orthopedic injury due to its frequently traumatic or acute nature and resulting substantial unplanned hospital expenses.

There is also a necessity for comprehensive studies addressing loss of work and productivity as related to seeking orthopaedic care. Current literature demonstrates that although long-term disability is a common outcome of injuries in sub-Saharan Africa, research is limited. Semi-structured interviews of 35 patients admitted to Uganda’s Mulago Hospital due to fractured lower extremities revealed anxiety over economic burden and disability from injury [18]. Interviews of 20 patients recovering from lower extremity trauma at a tertiary hospital in Malawi showed patients had missed several months of work, suffered reduced income, and were forced to sell assets (including their homes) due to lost income [19]. To our knowledge, there were two prior studies analyzing traumatic injury and orthopedic disease at KCMC. Casey et al. studied patients presenting with traumatic injury to Emergency Department and reported 44.1% had extremity fractures and an average length of stay (LOS) of 9.5 days [20]. Premkumar et al. found that 71.3% of orthopedic in-patients had lower extremity fractures with an associated average LOS in the orthopedic ward of 13.5 days [13]. Neither study analyzed hospital costs or the duration of disability due to injury. We identified only two previous studies in Tanzania that assessed disability days post-injury [21,22], indicating a research gap and the necessity to study the burden of orthopedic disease in this country.

In the discussion, we compare our results from Tanzania with studies conducted in other countries in sub-Saharan Africa. We identified four studies based in sub-Saharan Africa that quantified the socioeconomic impact of injuries. All four studies used survey-based methodology: O’Hara et al. surveyed 64 lower extremity fracture patients at Mulago Hospital in Uganda [12], Mock explored the indirect compensatory mechanisms of injury in Ghana [23], El Tayeb et al. studied 441 persons with non-fatal injuries in Sudan [24], and Juillard et al. assessed disability status, ability to return to work and reduction in earnings among 127 Nigerian patients following RTAs [25]. A comparison of the findings of this and similar studies to those of our study can be found in Table 11.

**Table 11:** Comparison of Injury & Disability Studies in Sub-Saharan Africa.

	Moshi, Tanzania (Current Study)	7 states in Nigeria (Julliard et al. 2010)	Mulago, Uganda (O’ Hara et al. 2015)	Asante, Ghana (Mock et al. 2003)	Khartoum, Sudan (El Tayeb et al. 2015)

**Study population**	200 patients with orthopedic disease (clinic)	127 patients from road traffic accidents (community)	64 patients with lower extremity fractures (in-hospital)	1542 patients with injuries (community)	441 persons with non-fatal injuries (community)
**% of patients disabled post-injury during survey**	52.9%	67.6%	–	67%	–
**Mean disability days**	232.9	–	–	Urban: 31.4 Rural: 26.4	14.9
**% of patients with >30 days disability**	75.3%	19.6%	–	–	–
**% of patients who lost employment**	40.6%	–	47%	–	9.3%
**% of patients who lost wages**	86.7%	88%	–	83%	–

### Indirect Costs

#### Disability

The short and long-term impacts of orthopedic injuries on disability must be quantified to better inform policies towards rehabilitation programs and physical therapy, employment protection, and disability benefits. In our study, 52.9% of patients reported disability as a result of injury. This finding is similar to disability as a result of injury reported in 67.6% of road traffic accident survivors in Nigeria and 67% of post-injury patients in Ghana [23,25].

The reported mean (SD) and median (IQR) duration of disability in our study population was 233(470) days and 90(30) days, respectively. In a study that surveyed patients in Dar es Salaam, Tanzania who had suffered road traffic accidents, mean disability days were 49 ± 78 [20], while in Ghana the mean (SD) disability days due to injury were 31.4 (51.5) and 26.4 (46.3) in urban and rural areas, respectively [23]. In Sudan, a study demonstrated mean post-injury disability days as 14.9 with 25.8 average disability days due to road traffic accidents [24]. The markedly higher duration of disability reported in our study compared to other studies in sub-Saharan Africa may be explained an increased proportion of patients with permanent disabilities in our study. However, the study did not measure permanent or terminal disabilities, thus the proportion is unknown.

#### Employment and Income Loss

The quantification of lost employment and wages demonstrates the devastating socioeconomic impact of musculoskeletal injuries in LMICs. Our study demonstrated that 40.6% of patients lost their job due to their injury, while 3.4% had to change their job due to their injury. Similarly, in a study of patients with lower extremity injuries, O’Hara et al. demonstrated that 47% of patients were unable to return to full employment [12]. In comparison, El Tayeb et al. demonstrated that 9.3% of injured patients in Sudan were unable to return to work; this discrepancy may be explained by the greater proportion of upper limb injuries in the study based in Sudan [24].

Of disabled patients in this study, 86.7% patients reported an individual wage decrease. Similarly, 88% of patients following road traffic accidents in Nigeria reported an individual wage decrease [25]. In Ghana, 83% of injuries led to intra-family labor reallocation and 33% led to a loss of family income [23]. In Uganda, there was a cumulative 88% decrease in wages reported by patients at Mulago Hospital post-injury [12]. Our study demonstrates similar rates of post-injury job loss and wage decrease as other reported studies in the region.

### Access to Treatment & Direct Costs of Hospitals

In all, 73.7% of the patients (n = 179) in our study reported that their current injury healthcare costs are a catastrophic burden to them and their family. A popular insurance option in Tanzania is the Community Health Fund (CHF), a government initiated program. The study found that individuals on CHF were protected against catastrophic expenditure compared to non-members [26]. CHF documentation claims coverage for minor surgeries, but major orthopedic surgeries do not appear to be covered, which explains our study’s finding that having health insurance did not improve access to orthopaedic treatment. Our study demonstrates a necessity to study the existing role of insurance for orthopaedic injury treatment in Tanzania. While preventative diseases appear to be covered by the Tanzanian government insurance option, traumatic injuries, often associated with considerable cost, require increased coverage to improve access to treatment. In addition, our study showed no difference in insurance status for patients who were salaried or unsalaried, indicating that employment-based insurance was rare for most occupations.

## Limitations

Our exploratory study of orthopaedic disease in Tanzania has several limitations. The study is based at a single tertiary hospital. However, because KCMC is one of the largest orthopaedic hospitals in Tanzania, we believe it can be considered a reasonably representative sample for the purposes of this exploratory study. However, of note our study had both a significantly older population with significantly fewer occupations as farmers than the national Tanzanian population. Thus, in order to develop national conclusions, it would be important to expand the study to a more representative national study sample.

We collected the data at a single time point in order to capture patients at varying lengths of time following injury, making this study unique in its capture of patients with long-term disability and chronic injury. This does, however, make recall bias an inherent concern, especially for patients who were injured years ago. In addition, there is an inherent selection bias as only patients who presented to receive care at the orthopaedic clinic were analyzed; patients who remain disabled but do not seek care for a variety of reasons are uncaptured. As with many studies investigating socioeconomic burden in low-income countries, this study relied upon patient reported data. Ideally, self-reported data should be supplemented with confirmed independently verified records.

## Conclusion

In addition to direct hospital costs, this exploratory study investigates the indirect costs of orthopaedic disease in Tanzania, including loss of wages and productivity. This study adds to limited literature regarding socioeconomic burden of injury and disability in sub-Saharan Africa and is the first such study conducted in Tanzania. As we hypothesized, our findings are generally in line with prior efforts to quantify direct and indirect costs of injury in sub-Saharan Africa. We found that a high proportion of patients with orthopaedic injuries have perceived catastrophic expenditure, significant post-injury disability, job loss, and decreased wages. This study further highlights the necessity to improve access to treatment, cost of treatment, insurance coverage of orthopaedic treatments, and rehabilitation and benefits for disabled patients.
